# In Defense of “Physician-Assisted Suicide”: Toward (and Back to) a Transparent, Destigmatizing Debate

**DOI:** 10.1017/S0963180124000434

**Published:** 2026-04

**Authors:** Brandy M. Fox, Harold Braswell

**Affiliations:** 1 Center for Biomedical Ethics, Stanford University, Stanford, CA, USA; 2 Albert Gnaegi Center for Health Care Ethics, Saint Louis University, St. Louis, MO, USA

**Keywords:** physician-assisted suicide, medical aid-in-dying, physician assisted death, suicidism, disability rights, end of life

## Abstract

Many bioethicists have recently shifted from using “physician-assisted suicide” (PAS) to “medical aid-in-dying” (MAID) to refer to the act of voluntarily hastening one’s death with the assistance of a medical provider. This shift was made to obscure the practice’s connection to “suicide.” However, as the charge of “suicide” is fundamental to arguments against the practice, “MAID” can only be used by its proponents. The result has been the fragmentation of the bioethical debate. By highlighting the role of human agency—as opposed to natural processes—in causing death, the term “PAS” makes it easier both to perceive potential risks to vulnerable populations and to affirm suicide as a potentially autonomous choice. As such, “PAS” thus more transparently expresses the arguments of both supporters and opponents of the “right to die,” while avoiding the unnecessary stigmatization of suicide and suicidal people which is a result of the usage of “MAID.”

## Introduction

Contrary to its title, this article is not a defense of physician-assisted suicide. Nor is it an attack on the practice. In fact, it presents no opinion on the rightness or wrongness of physician-assisted suicide. What it does present is an opinion on the correct terminology with which to refer to the practice of voluntarily hastening the end of one’s life with the assistance of a medical provider. Our opinion is that the term that bioethicists should use to refer to this practice is, yes, “physician-assisted suicide.”

A few years ago this would not have been an argument that one would have to make— indeed, it would have made no sense to do so. In the United States, and elsewhere, supporters of physician-assisted suicide referred to the act as such.^1,2^ Indeed, they even advocated for doing so, so as to distance the act—both analytically and in the popular imagination—from “euthanasia,” a term many associated with Nazism.^3,4,5^ Physician-assisted suicide was the dominant term used in discussions about the practice, including those concerning the Oregon Death with Dignity Act.^6,7^ This term was shared in general by both supporters and opponents of the practice—marking a rare truce in an otherwise contentious debate.^8^ Indeed, this truce was the foundation of debate, for it established a consensus with regard to the very thing being debated. That “thing,” everyone understood, was physician-assisted suicide. All parties agreed that “causation and human agency [are] not in dispute,”^9^ allowing room to discuss the ethical implications of the act.

But this situation has changed dramatically. Supporters of physician-assisted suicide (PAS) began replacing the term with a variety of other ones: aid-in-dying (AID), medical aid-in-dying (MAID), and assisted death (AD).^10,11,12^ These three terms (AID, MAID, AD) are used to cover a variety of practices, depending on the jurisdiction the act takes place in. For example, in Canada, there are two types of MAID: A physician or nurse practitioner can either administer a substance that causes death, or (s)he can provide a drug that the patient then self-administers.^13^ The first action is also known as euthanasia; scholars have argued that this bundling of terms is confusing in itself.^14^ The only form of “aid-in-dying” legally available in the United States is for the medical provider to prescribe a substance to a terminally ill patient, who then ingests the substance with the intention of causing death.^15^ These types of “aid” can also be applied to different populations: in the US, for example, this assistance is only accessible to people who are dying. In Canada it is available to people with long-term disabilities; such people are not “dying” in the sense that the term is generally used, yet they are eligible for “assistance in dying.”^16^

What united these new disparate terms (AID, MAID, AD) was their common exclusion of one word: suicide. This exclusion was in large part politically motivated, as PAS supporters found that removing reference to “suicide” increased support for the practice.^17,18,19,20^ But supporters of the new terminology have also defended it on analytic grounds, arguing that there are valid philosophical reasons for excluding suicide from the definition of the act.^21,22,23^ The effect has been that both the pro- and anti-PAS factions have further stigmatized the term “suicide” by emphasizing and perpetuating the idea that suicide should never be an option.

In this article, we argue against those reasons for excluding the term suicide. We begin by noting the radicality of the shift from “PAS” to “MAID.” While “PAS” refers to a death that is caused by human action, in “MAID” death is a fact of nature. The result is not simply a newer, more politically expedient term for the same phenomenon. It is a redescription that transforms the very nature of the phenomenon being discussed.

This redescription has been broadly accepted by “right to die” proponents. However, opponents, in contrast, have rejected it, generally still referring to the phenomenon being debated as “PAS.” This rejection is not coincidental. The premise of opponents’ argument is that the “right to die” represents a discriminatory double standard in the treatment of suicidal ideation. It is impossible to make such arguments if the phenomenon being debated is not classified as a form of “suicide.” The very terms of “debate” render the position of PAS opponents fundamentally excluded. The result has been the fragmentation of the “right to die” debate into two camps that do not agree on the basics of what is being discussed.

This fragmentation is, we believe, wholly unnecessary. PAS, we contend, is a term that, precisely because it highlights the role of human agency in causing death, more effectively expresses the best arguments for and against the practice. It highlights the potential risks to vulnerable populations of having their lives ended in a manner both violent and premature. Also, it also emphasizes the possibility that the decision to die can be an autonomous choice. Both these possibilities are, at best, muddled—and at worst obscured—by the usage of “MAID.” The term has thus diverted the course of the “right to die” debate, even as it undermines both of the positions that had previously given the debate structure. The result, in the words of the American College of Physicians, is to “obscure[ing] the ethics of what is at stake.”^24^ This obfuscation should be rejected by both sides of the debate, as well as by the field charged with giving that debate structure: bioethics. For these reasons, we advocate a return to “PAS.” We begin by clarifying the nature of the shift that has taken place.

## Two different things

Sam, a man with a terminal medical condition, goes to his doctor. Sam asks the doctor to prescribe a substance. The reason Sam wants this substance is because taking it will bring about the end of his life. The doctor knows this, and that is the reason why she prescribes it. Sam acquires the substance and later ingests it. He dies in a manner that is directly traceable to his having done so.

Sam’s action—his taking of this substance—can be described in two different ways. One is “physician-assisted suicide” (PAS). The other is “medical aid-in-dying” (MAID). The difference between these terms is seemingly semantic; they appear to be two different terms to describe the same thing. However, that is not the case. On the contrary, “PAS” and “MAID” describe two things that are radically different, even fundamentally opposed. Rather than describe Sam’s action, they splinter it into two separate actions that are fundamentally irreconcilable.

In “PAS,” Sam is a person who wants to die: his foreseeable future is unacceptable, and he wishes to end the possibility of that future before it is upon him. He is *suicidal*. When Sam tells the doctor that he wants to die, he confesses this suicidality. However, the doctor treats this suicidality differently than she would otherwise. Rather than discourage Sam, she prescribes him a substance that Sam can take to end his life. When Sam does so and subsequently die, that substance is the cause of his death, as is—somewhat more distantly—Sam himself. By prescribing Sam this substance, the doctor has “assisted” Sam’s suicide, though she has not directly caused it.

In “MAID,” Sam is not suicidal. He is *dying*. If he could, he would live. However, he will die. He merely wants to die *sooner* than would otherwise be the case. The substance the doctor prescribes him will bring about this death more quickly. However, this medication will not cause Sam’s death, nor will Sam himself. Rather, the cause of death is Sam’s disease. The prescribed substance merely “assists” it. Sam does not die of suicide. He dies a natural death.

These two actions bear no relation to each other. “PAS” is a death that is voluntary, medicalized, and caused by human action. “MAID” is an involuntary death, provoked by nature. In their relationship to death, PAS and MAID are opposite. The difference between them is semantic. However, here, semantics is destiny. This destiny does not just refer to the nature of the act itself. It also refers to the debate that can emerge around it. We will now describe how this debate has changed with the shift from “PAS” to “MAID.”

## A good debate…

For a significant portion—perhaps most—of the past thirty years, the term used to describe Sam’s action would be “PAS.” This description was broadly agreed upon. However, this agreement on “PAS” did not mean that there was nothing to argue about. On the contrary, the agreement about the term “PAS” made possible a debate about the act of PAS.

PAS supporters thought that PAS was a form of suicide that could be justified. PAS opponents did not. Underlying this disagreement were questions both empirical and principled. For example, empirically, was the suffering attested to by individuals seeking PAS due to incurable medical conditions or to social contingencies that could be remedied via means less radical than the provocation of death?^25,26^ With regard to principles: Was self-killing always bad or could, in some instances, it be considered to be an act that was morally neutral or even laudatory?^27,28,29,30,31,32^

Both these empirical and principled questions were reasonable to disagree about. However, such disagreement would have to be attuned to the contingent nature of the topic in question. This was not just a matter of updating one’s data—however important—while leaving one’s principles untouched. Different data could impact the matters of principle, calling into question, for example, which position on PAS really supported “autonomy” or the “sanctity of life.”^33,34,35,36,37,38,39^

The result was a debate that endured for at least 30 years, and arguably much longer. Not only endured—but *improved*. Participants in the PAS debate had, over time, to take into account a broader range of views that both opposed and supported their own. Over this time the debate became more interdisciplinary and interprofessional, drawing on resources across academic, legal, and health professions. It also came to contain a far broader range of political, religious, and philosophical perspectives than before.^40,41,42,43^

These perspectives themselves became, with time, more nuanced and robust. Viewpoints that once seem relatively unified have revealed themselves to be sites of rich contention: For example, while the debate was largely split along secular/religious lines, there emerged secular arguments against PAS and religious ones for it.^44,45,46,47,48,49^ This was true of any number of communities in the debate: feminists, disability rights activists, hospice professionals, etc. The PAS debate thus spawned a multitude of additional debates, each one of which enriched the larger discussion.

The result was not just a good debate. It was a model for what debate should be. This model reflected well on its participants: both the advocates and the opponents of PAS, the various philosophical and political groups engaged in it, and the field of bioethics, which acted as a self-appointed curator of the discussion, setting its basic terms. Ultimately, it reflected well on the societies in which it took place, and on the institutions of higher learning responsible for the scholarship that informed it. It was a debate that realized the larger goal of the university and even participatory democracy itself.

That debate is now distracted. Neither side has “won” the debate, at least not as that term might be conventionally understood. The issues underpinning this debate have not been resolved. However, they have been pushed from view. Indeed, the very framework that is now used to discuss the phenomenon being debated renders several of the debate’s important philosophical tenets fundamentally invisible. The name of that framework is “MAID.”

## …And its fragmentation

It is tempting to view the term “MAID” as another worthwhile innovation in the PAS debate. This is the argument of proponents of the term, who claim that “MAID” or “PAD” more accurately represents the action it describes.^50,51,52^ But the effect of “MAID” is not to improve the “right to die” debate. This result is inherent to the term itself.

“PAS” describes an act that could be debated. The reason why one could debate this act is because it was justifiable to agree or disagree about it. Participants in the debate could do so from either perspective without betraying their argument or their underlying principles. They could disagree on the merits of the act because they could all agree about the matter they were disagreeing about: that matter was suicide. “MAID” erases suicide from the discussion. This is, according to its proponents, the term’s purpose. Regardless, it is its effect. “MAID” transforms suicide into a natural death, shifting its cause from human behavior to underlying illness. One could debate whether this transformation accurately describes the phenomenon in question—and we will. However, before doing so, we want to highlight how adopting this term transforms not the act but rather the ethical debate about it.

When using the term “MAID,” it is possible to support the act. However, it is not possible to oppose it. If we were to agree that what is “really” happening in Sam’s case, described above, is a natural death hastened but not caused by human action, then there would be no cause for disagreement about whether that death was good or bad. His “natural death” would be an unquestioned good.^53^ PAS opponents have never claimed to oppose hastening death by an action so negligible that it does not disrupt an underlying natural process; their claim is that the action, in this case, causes a disturbance so significant that it takes dying out of the realm of nature and into that of human causality. These arguments—all of them—begin with the premise that PAS is a form of *killing*.^54,55,56,57,58^ “MAID” begins with a premise that is precisely the *opposite* of this: the sequence of events is *not* killing. Also, this is a premise that opponents of the act under discussion cannot accept. To do so, they would have to support the act. Opposition to self-killing is the core objection of PAS opponents. If the act being discussed is *not* killing, but rather a “negligibly hastened natural death” (the description implicit in MAID), then there is nothing to oppose.

One might argue against this point by saying that MAID adheres to a logic that is similar to that of the so-called “principle of double effect,” (PDE) which many opponents of PAS support. Like MAID, the principle of double effect justifies calling a death “natural” even if it was caused by a human—and, more pointedly, medical—action.^59,60^ It makes it possible, for example, for a doctor to give Sam a dosage of morphine that both relieves his pain and ends his life without being accused of killing him. How is this different, in its underlying logic, from calling deaths that result from MAID “natural?”

It is different because the justification for the principle of double effect is *intention*. PDE proponents argue that intentionality determines the nature of action. In the end-of-life scenario described above, Sam’s death can only be classified as “natural” if the action that provoked it was not intended to do so. For example, if the reason the physician gave Sam the morphine was primarily to alleviate his pain—with no intention of ending his life—then the principle of double effect would be satisfied. If the doctor’s intention was killing, then it would not, and the death would be considered the result of human action. This is different from MAID. The primary intention behind the administration of MAID is to end Sam’s life. The determination regarding whether the resulting death is “natural” or not is therefore not based on intention. “MAID” is based on an implicit interpretation of the relationship between nature and time: that there is some period of time—presumably six months or less, the duration of the terminally ill individual’s prognosis—by which an individual (Sam) can have his life deliberately shortened by a health care professional without that shortening “causing” death. The underlying logic used here is wholly different than that of “double effect.”

There are thus no grounds on which opponents of the “right to die” could participate in a debate about “MAID.” From some opponents’ perspective, as Kevin Yuill has put it, “to call what happens in Oregon and Washington… anything other than suicide is patently ridiculous.”^61^ He is not the only one who has made this argument.^62,63,64^ This conclusion can also be supported by empirical evidence. In Sheri Gerson’s study of hospice professionals’ understanding of the difference between MAID and suicide, though many healthcare providers recoiled at the word “suicide”, one physician stated, “I do not think it helps us to pretend that our patients are not actively taking their own lives.”^65^

But the field of bioethics has largely moved to “MAID,” and this move’s impact has been profound. The result is, instead of one debate, we now have two separate “right to die” discussions. The terms framing each discussion—“PAS” and “MAID” respectively—are no longer primarily, or even relatively, descriptive; they are indicators of the normative assessments of the individuals using them. PAS opponents use “PAS” while MAID (or PAS) supporters use “MAID”—assessing sympathetically identifying with others who use whatever one considers to be the “right” term, and disregarding everyone else. This is neither thought nor intellectual exchange—and it does nothing to advance our understanding of what remains, in our view, an important issue that continues to be worth discussing.

In such an environment—in which the term one uses is taken, often accurately, as a window into one’s ethical analysis—it might seem a foreordained conclusion that we, advocates of the term “PAS,” oppose PAS the practice. This is not true: one of us opposes it and the other supports it. However, such possibilities, once common, are now difficult to conceive. This difficulty is not one that should be celebrated by “right to die” supporters. On the contrary, the loss of “PAS” has effaced pro-PAS arguments that are worthwhile in themselves, different from those put forward for MAID, and, in our opinion, significantly stronger. A return to “PAS” is thus not one that benefits only, or primarily, PAS opponents. It is better for everyone.

## Why “PAS” is better for everyone

As we have tried to emphasize throughout, the shift from “PAS” to “MAID” is not a small one. It is fundamental, as are the implications that it has for long-standing aspects of the “right to die” debate. Here we focus on its implications for what we consider to be the most central—or certainly *among* the most central—concerns of both PAS supporters and opponents: the protection of vulnerable populations and the exercise of individual autonomy. Although these concerns are often pitted against one another—with there being a presumptive tension between the affirmation of suicidal autonomy and the protection of vulnerable populations—here we find that the term “MAID” damages both of these concerns. For this reason, the “MAID” terminology undermines arguments both in favor of and opposed to the “right to die.” It is from a shared acknowledgment of the validity of “PAS” that one can most effectively support or oppose the act itself.

### Protection of vulnerable populations

The strongest argument, in our opinion, against PAS has come from advocates for disability rights. This argument considers PAS to be a discriminatory double standard in the application of suicide prevention: while suicide is condemned in able-bodied people, people who are terminally ill and, in some cases, chronically sick and disabled are denied routine interventions to alleviate suicidal ideation. This is a double standard, and it is *discriminatory* because it is based on the assumption that the lives of terminally and chronically ill people are worth so much less than able-bodied people that they are arguably not worth living.

This discriminatory double standard would be bad enough in the abstract. However, it does not occur in the abstract. It occurs, rather, in the context of a society that is already explicitly and implicitly discriminatory against terminally and chronically ill people. This discrimination includes the denial of routine services, physical segregation in institutions or negligent home environments, and ceaseless devaluation of cultural products.^66,67,68,69,70^ This broader *social* discrimination has been shown to drive terminally ill and disabled people to want to take their lives.^71,72,73^ But PAS, rather than recognizing the discrimination underlying suicidal ideation, either ignores it or, arguably, affirms the bias.

Opponents argue that PAS is thus not just bad in the abstract. It is bad because it furthers existing systemic discrimination of the sort that drives terminally ill individuals into the “forced choice” to end their lives.^74,75^ PAS, thus, is a discriminatory double standard in the treatment of suicidal ideation in a group of people who experience precisely the sort of discrimination that drives people to suicide.

“MAID” does not alleviate this double standard. On the contrary, it exacerbates it. It creates two categories of suicides: one, which is described as a “suicide”; and another which is considered a “natural death.” The effect of this distinction is to make it almost impossible for those terminally ill—and potentially chronically disabled—people who seek “MAID” to complete suicide. This excludes them from suicide prevention, an exclusion which, in itself, is discriminatory. Also, it is additionally discriminatory in its implication that their lives are of so little value that they cannot complete suicide, to begin with. Determining that their suicides were “rational” might carry a similar implication,^76^ but it would be notably less severe than the denial that they had completed suicide at all.

Excluding the word “suicide” from describing the act of hastening one’s own death also contributes to the stigmatization of suicidal people. Strict avoidance of the word “suicide” furthers a specific type of discrimination identified by Alexandre Baril as “suicidism.” Suicidism is “an oppressive system (stemming from non-suicidal perspectives) functioning at the normative, discursive, medical, legal, social, political, economic, and epistemic levels in which suicidal people experience multiple forms of injustice and violence.”^77^ One of suicidism’s primary operations is to promote “compulsory aliveness,” a vitalist view that values continuing life above all else.^78^ We stand with Dr. Baril and include the word “suicide” in the act of PAS because we wish to add our voices to the project of reclaiming “suicide” from entirely negative connotations and allow alternative narratives about the state of wanting to bring about one’s own death sooner than that death would occur naturally to be heard.^79^ It is possible for a suicidal act to be undertaken as an assertion of autonomy, the most extreme declaration of liberty, or as a response to oppression.^80,81^ We wish to include the word suicide to reclaim and re-emphasize these myriad of meanings.

In contrast to suicide, “MAID” erases the role of human action in the provocation of death. Since discrimination would, presumably, come through human action, this makes it much more difficult to examine the potential for bias. This is true of both proximal discrimination—such as that coming from the suicidal individual, as well as the health care workers and family members they interact with—as well as distal discrimination, grounded in the structure of society. One can note the possibility and even the presence of such discrimination while supporting PAS; indeed, this was in part the premise behind the arguments of disability rights advocates, like Andrew Batavia and Lennard Davis, who have supported the practice in the past.^82,83^ But “MAID” makes it much more difficult to *acknowledge* these factors, since doing so requires recognizing that the individual *wants* to die to begin with. “MAID” erases this desire, and its key role in the causation of death, under the cloak of nature. The cause of death in such circumstances is listed as the terminal health condition.^84,85^ This seems particularly ironic, considering that patients chose PAS precisely to ensure that they did not die from their disease. With the inclusion of the word suicide, we wish to center the intention and agency of those who wish to hasten their own death.

“MAID” thus carries immensely greater risks than “PAS” for vulnerable groups and those who are concerned about protecting them. Perhaps such risks could be justified were “MAID” to dramatically expand individual autonomy. However, in this respect also, “PAS” is a much better term.

### Valuing autonomy

There was a time in which PAS advocates argued that suicide was a fundamental right. PAS, in this view, was the epitome of the individual’s control over her life, a control that, naturally, would culminate in the decision to bring—or not—that life to an end. Suicide can be seen as a radical re-orientation against a world where one’s preference not to live is interpreted as inherently irrational and pathologized. Suicidal individuals are often stigmatized and oppressed. By re-inserting the word “suicide,” we seek to unlink the act of voluntarily ending one’s life from “pathologization, alienation, [and] stigmatization” that is rampant under the current regime of suicidism.^86^ PAS was not previously just another instance of “patient autonomy.” It was *the* instance of autonomy itself: a special instance, one that represented an underlying idea of freedom that defined human progress and even human nature itself.^87,88,89,90,91^ No more. PAS was the apex of autonomy. MAID is an act of nature. Unlike physician-assisted suicide, MAID does not describe an individual act of self-killing. It describes instead a culmination of a biological process: dying. Human action—the prescription and ingestion of a fatal substance—does not alter this process.^92^ The action facilitates death, working with nature rather than against it.^93^ MAID does not then refer to a human-driven act; it is an act of nature.

MAID is thus not, contrary to the claims of its proponents, a “choice.” It is, rather, a capitulation: a recognition that there are, in fact, harsh limits on human choice and that the choice to die is the most fundamental among them. MAID does not thus give individuals the “freedom” to die. Rather, it indicates that they never had this freedom. In the process, it implies that the desire *to have* this freedom—the desire to die—is morally wrong: that suicide, in itself, is inherently bad. This places a hard limit on autonomy while furthering the stigmatization of those people—suicidal people—who would seek to surmount it. Also, it diminishes the choices made by those who seek “MAID”: it strips them of agency over their deaths.

However, suicide *can* be an autonomous choice. This, at least, was the argument of those who supported PAS. Also, this argument tied this support to PAS to a larger conversation about the ethics of rational suicide itself. This was intellectually worthwhile: suicide is worth debating, and PAS should be a part of that debate. Also, it was something that both supporters and opponents of PAS could appreciate. Indeed, one could even accept that PAS furthers autonomy while opposing the act—a position that is impossible in the era of “MAID.”

Ultimately, “PAS” makes it possible for those who seek it to affirm their choice to die as the fullest expression of themselves. “MAID” significantly weakens their ability to do so, and, in the process, limits the extension of human autonomy itself. In the process, it stigmatizes suicidal individuals, who, from the perspective of “MAID,” continue to use their autonomy in a manner implicitly irrational and unethical.^94,95,96^ The term “MAID” should thus be opposed, on analytic grounds, even by “right to die” supporters. The best justification for the term is not analytical but political: it makes it easier to garner support for what is in reality PAS. It is this political argument that we address in our conclusion.

## Conclusions

We want to return to Sam. If we describe Sam’s action as “PAS,” it opens up a range of possibilities: the possibility that he might have been driven to suicide by discriminatory social factors that could be alleviated via means much less drastic than a self-inflicted death, as well as the possibility that his desire to die is a rational reflection of his will and that he is justified in taking his life. These possibilities could be explored and, based on one’s findings, could inform the normative conclusions that one might draw about both Sam’s particular act of suicide as well as the larger topic itself.

But “MAID” opens up no such possibilities for Sam—or anyone. “MAID” strips him of his choice to die. It also strips him of the basic protections provided by the ability of society and those involved in his death to scrutinize what drove him to make this choice. Indeed, there is no “choice” to scrutinize because Sam’s death, as an example of “MAID,” is a manifestation of nature itself. His death, as a result, means nothing, except that nature was, in the end, in control. However, this is not true: nature was not in control in Sam’s death. Sam was. Also, this acknowledgment can and should form the basis of any argument in favor of his having such control. This can be true for defenses of PAS. However, we also think that it provides a foundation for the best defense of the term “MAID.”

The best argument in favor of “MAID” is that it is the term that, politically, can do the most to extend the control that dying people have over the ends of their lives. This is because to use the term “suicide” to refer to Sam’s action would make the creation of a legal and medical context in which he might take such an action significantly more difficult, perhaps particularly in the United States today. This is the *political* argument for “MAID,” one that supporters of the term have often considered to be secondary, but that we believe to be superior. In this political argument, the overall good that would come from legalizing PAS under the rubric of “MAID” would overwhelm any objection to the use of inaccurate or misleading terminology. This is a compelling point if one believes—as both PAS and “MAID” supporters do—that giving individuals the right to die is a significant, perhaps even fundamental, good. The usage of “MAID” has almost certainly built the political support necessary to extend access to PAS to millions of people. It thus, in theory, does a great good and one might even argue that using “MAID,” even misleadingly, in such a context becomes a moral necessity in that it makes this good possible.

One could further this point by noting that in the public sphere, opponents of PAS often have seemingly very little qualms with making misleading arguments about the motives of PAS supporters and the impact of the practice itself. Politics is, as the saying goes, a dirty game. Also, it is not fair to hold PAS supporters to a standard that is significantly higher than that of PAS opponents, indeed, one that might make their cause difficult if not impossible to achieve.

We disagree with this argument. We believe that it is not practically impossible to garner significant public support for PAS, and that doing so, in fact, might even have residual benefits—such as provoking a broader, less stigmatizing conversation about the ethics of suicide and creating a greater awareness of disability rights—that should figure into any ethical calculation. We also believe that transparency in political discourse is a worthwhile virtue and that betraying it comes with costs that, though seemingly subtle, can be quite profound. We think that the overall benefit to PAS supporters, opponents, and, particularly, those undecided on the practice, of using “PAS” outweighs the instrumental value of the move to “MAID.” And we think that we owe at least this much to Sam: to be candid with him and to recognize and honor—perhaps, even in disagreement—the choice *he* made.

We believe this—and we may be wrong. However, we at least want someone to *tell us* that we are wrong, and to do so in a scholarly manner befitting of bioethical debate. It is that lack of such arguments—arguments in favor of the term “MAID”—in bioethics journals that we find particularly concerning. PAS and MAID are topics pertaining to one of our field’s core issues—euthanasia—and the need to thoroughly debate them is equivalent to a *raison d’etre*. That the move to “MAID” has occurred without any rigorous debate is alarming, for it undermines the putative justification for bioethics itself. However, unlike some of the other effects of this shift, this consequence can be remedied, and it is partly with that goal in mind that we have written this article.
